# Distribution of *Plasmodium falciparum* gametocytes and malaria-attributable fraction of fever episodes along an altitudinal transect in Western Cameroon

**DOI:** 10.1186/s12936-015-0594-6

**Published:** 2015-02-26

**Authors:** Timoléon Tchuinkam, Bridget Nyih-Kong, François Fopa, Frédéric Simard, Christophe Antonio-Nkondjio, Herman-Parfait Awono-Ambene, Laura Guidone, Mbida Mpoame

**Affiliations:** Malaria Research Unit of the Laboratory of Applied Biology and Ecology (MRU-LABEA), Department of Animal Biology, Faculty of Sciences of the University of Dschang, P. O. Box 067, Dschang, Cameroon; Hôpital Saint Vincent De Paul, Mission Catholique Sacré Cœur, BP 011 Dschang, Cameroon; Laboratoire de Recherche sur le Paludisme, Organisation de Coordination pour la lutte contre les Endémies en Afrique Centrale (OCEAC), BP 288 Yaoundé, Cameroun; MIVEGEC, UMR IRD224-CNRS5290-UM, Institut de Recherche pour le Développement, 911 Avenue Agropolis, BP 64501, Montpellier, France

**Keywords:** Highland malaria, Infectious reservoir, Fever episode, Malaria early warning system, Epidemic

## Abstract

**Background:**

Highland areas are hypoendemic zones of malaria and are therefore prone to epidemics, due to lack of protective immunity. So far, Cameroon has not succeeded in implementing a convenient and effective method to detect, prevent and forecast malaria epidemic in these peculiar zones. This monitoring and evaluation study aims to assess the operational feasibility of using the human malaria infectious reservoir (HMIR) and the malaria-attributable fraction of fever episodes (MAFE) as indicators, in designing a malaria epidemic early warning system (MEWS).

**Methods:**

Longitudinal parasitological surveys were conducted in sentinel health centres installed in three localities, located along an altitudinal transect in Western Cameroon: Santchou (750 m), Dschang (1,400 m) and Djuttitsa (1,965 m). The syndromes of outpatients with malaria-like complaints were recorded and their blood samples examined. The HMIR and the MAFE were estimated and their spatial-temporal variations described.

**Results:**

The prevalence of asexual *Plasmodium* infection in outpatients decreased with increasing altitude; meanwhile the HMIR remained fairly constant, indicating that scarcity of malaria disease in highlands is likely due to absence of vectors and not parasites. In lowland, children carried the heaviest malaria burden in the form of febrile episodes, and asexual parasites decreased with age, after an initial peak in the 0-5 year’s age group; however, they were similar for all age groups in highland. The HMIR did not show any variation with age in the plain; but some discrepancies were observed in the highland with extreme age groups, and migration of populations between lowland and highland was suspected to be the cause. *Plasmodium* infection was perennial in the lowland and seasonal uphill, with malaria disease occurring here mostly during the short dry season. The MAFE was high and did not change with altitude.

**Conclusion:**

It is obvious that a malaria outbreak will cause the sudden rise of HMIR and MAFE in highland, prior to the malaria season; the discrepancy with lowland would then help detecting an incipient malaria epidemic. It is recommended that in designing the MEWS, the National Malaria Control Programme should include these parameters and put special emphasis on: altitude, age groups and seasons.

## Background

In the lowland regions of sub-Saharan countries, malaria is holoendemic with perennial transmission. On the contrary, highland areas are known to be malaria hypoendemic, due to climate (low temperature and relative humidity), which is not appropriate for anopheline development and their reproductive fitness [1]. Nevertheless, the success probability of an entomological inoculation in these regions of low transmission is higher than in holoendemic areas [2,3]. In fact, the highest disease risks are observed among populations exposed to low-to-moderate intensities of transmission, and mean age of disease patients increases with decreasing transmission intensity [4].

An epidemic is a sharp increase in malaria incidence among populations in which the disease is scarce; or a sudden high increase in clinical malaria for areas of moderate transmission [5]. For the past decades, there has been an increase in the number of malaria epidemics, and the question is raised of a boosting of malaria transmission in African high altitude areas as a consequence of global warming [6-8]. It was estimated that 110 million people are at risk of malaria epidemics in Africa and 110,000 of these die of the disease each year [5]. Another survey indicated a higher rate of 155,000–310,000 deaths (out of 12 million malaria episodes) attributable to epidemics [9]. Unlike the lowland environments where the potential for malaria epidemics, due to decreasing levels of natural immunity, may be offset by negative impacts of urbanization on anopheline mosquito larvae [10], control strategies in highland areas should be much more based on prevention of epidemics. Tools to predict and forecast malaria epidemics are, therefore, needed.

Recently, modern tools to help decision-makers to predict malaria epidemics were proposed. They rely on the use of: combinations of satellite-derived climate data [11,12], weather monitoring combined with disease surveillance [13], the normalized difference vegetation index (NDVI) [14], the El Niño Southern Oscillation phenomenon (ENSO) [15,16], and soil moisture [17]. Guidelines for implementing some of these models were provided and approved [18,19]; they defined the steps to take while setting up a national malaria early warning system (MEWS). Unfortunately, very few African countries have adequately qualified human resources and appropriate meteorological facilities to be able to implement such measures. Consequently, the challenge of developing new tools for malaria prevention in highlands remains.

Since malaria epidemics appear suddenly and terminate within a few months, they require emergency measures that must be implemented as promptly as possible in order to be effective. Moreover, a declaration of an emergency, generally based on the inability of the medical services to cope with demands of patients following malaria outbreaks, must be made. Forecast of epidemics based on indicators preceding elevated infection and disease rates in human populations are required. Such a strategy is likely to be efficient, since there is an elevation of gametocyte prevalence, which can reach 6% in the general population and even more (8 to 10%) in infants, prior to malaria epidemic [20]. In this respect and in the absence of the above-mentioned sophisticated tools, network of health workers in sentinel stations should be installed in highlands to monitor epidemiological, social and environmental factors. It has been shown that this system can also play a role in predicting the timing and severity of malaria epidemics [21-23], and that it is likely to be cost-effective [24]. Unfortunately, the National Malaria Control Programme in Cameroon have neither designed nor implemented a convenient and effective method to detect, prevent and forecast malaria epidemic in these particular zones [25].

Malaria infection tends to be a disease when a defined level of parasitaemia leads to the production of signs and symptoms [26-28]. Malaria disease is characterized by syndromes among which fever is the most prominent symptom, but also a common manifestation of many infectious diseases. For quite some time, fever has been a subject of studies and comments; yet it is still a matter of controversy, as whether it has a beneficial adaptive value, or is just a harmful trivial side-effect in the host response to infections [29]. Conclusive evidence is still lacking that it has survival value and may be beneficial. According to some authors, non-malarial fevers can suppress low levels of parasitaemia through cytokine production [28]. Moreover, fever itself is a natural mechanism of defense, because it raises body temperature, increases the metabolic activities and makes the immune response more efficient [29-31]; it may, therefore, hinder the development of the parasite [32]. Conversely, others say fever can reduce the individual immunity and the low parasitaemia will be quickly boosted to reach the pyrogenic threshold and thus clinical malaria. The question raised in this study is whether fever could be an indicator of disease trends and hence be useful in detecting, preventing and forecasting malaria outbreaks.

Because fever is present in the majority of malaria cases in sub-Saharan Africa, it has been recommended that for all febrile symptoms, a presumptive treatment should be administered [33]. The approach was of value to clinicians, but not to epidemiologists. In fact, it is not clear so far, whether the presumptive treatment of fever episodes as malaria cases is a help or a hindrance for malaria control [34]. Recently, with improvements in the rapid diagnostic tests, that enable quick and easier examination of blood, WHO moved away from presumptive treatment to one that recommends parasitological diagnosis whenever possible [35]. The aim of this shift of policy was to avoid treating a large number of non-malarial fevers, which represent a non-negligible fraction in endemic areas, in contrast to highlands. The potential benefits of parasitological diagnosis will depend mainly upon the prevalence of *Plasmodium* infection (i.e. the *P. falciparum* parasite rate (PfPR)), among patients who report fever, knowing that these malarial fevers are always associated to other manifestations [36].

Although fever is the main characteristic sign of malaria, some *Plasmodium* infection cases do not present measurable temperature elevation [28]. These asymptomatic malaria cases remain a challenge for malaria control programmes, as they significantly influence transmission dynamics. The crucial gaps in the knowledge of asymptomatic malaria that should be the focus of future research towards development of more effective malaria control strategies have been highlighted [37]. Asymptomatic carriage of malaria parasites occurs frequently in endemic areas, and the detection of parasites in blood from a febrile individual does not necessarily indicate clinical malaria [28]. In fact, the high prevalence of asymptomatic malaria infections and the non-specificity of signs and symptoms of the disease, make the individual diagnosis of clinical malaria uncertain in highly endemic areas [27,38]. However, in areas of low endemicity like in highlands, the presence of parasites in fever cases can be attributed to malaria [28]. The human malaria infectious reservoir (HMIR) (i.e. the prevalence and density of sexual parasites), as well as the malaria-attributable fraction of fever episodes (MAFE) (i.e. the PfPR taken among the patients with malaria-related complaints), should be given special considerations in the epidemiology of highland malaria.

The hypothesis of this study is that in normal circumstances, HMIR and MAFE in lowland and highland areas are not different, since the higher number of infective bites found in lowland will result in only low infection in human populations due to naturally acquired immunity; while in highlands, although there is no immunity, there are also low infective bites, because the climate is unfavourable to the *Plasmodium* extrinsic cycle, the anopheline development and reproductive fitness. Moreover, this absence of immunity may boost the trophozoite-gametocyte transformation ratio (TGR) and extend the longevity of sexual parasites. It is, therefore, likely that early increases in HMIR and MAFE in sentinel health facilities of highland areas will precede the sudden elevation of clinical malaria disease incidence. Thus, monitoring these two parameters using network of health workers in sentinel stations placed in highlands may unveil incipient epidemic and provide good conclusive early warning of malaria outbreaks.

The main objective of this study was to evaluate the operational feasibility of using the HMIR and MAFE as indicators to be used by a network of health workers in sentinel stations, while designing a MEWS for an epidemiological surveillance of malaria outbreaks.

## Methods

### Study areas

**Figure 1:**
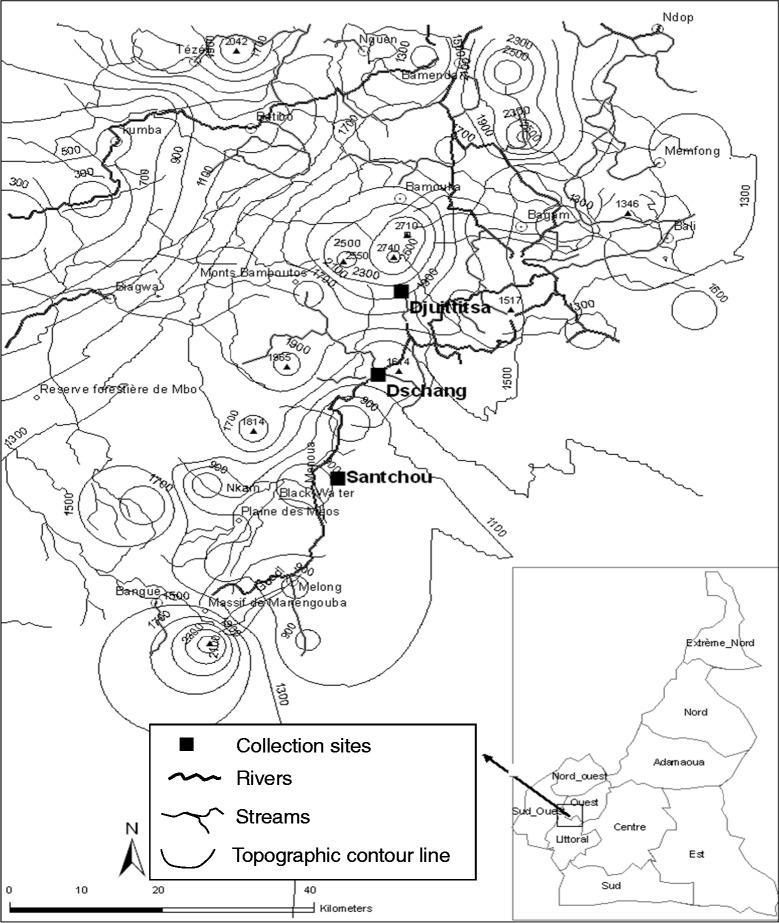
**Topographic map of the Menoua Division of Cameroon showing the geographical localizations of the collection sites.**

The study took place in the Menoua Division, an outskirt of the Cameroon’s Western highlands, named the Bamileké Plateau. It is a unusual zone as far as topography and climate are concerned, located on the Cameroon Volcanic Line in a savannah landscape within the Guineo-congolese bioclimatic domain as follows: the main dry season (MDS: November to mid-March), the small rainy season (SRS: mid-March to May), the small dry season (SDS: June to July) and the main rainy season (MRS: August to October) [39]. The major malaria vector species in this zone are *Anopheles gambiae* and *Anopheles funestus* [40]. About 97.30% of human malaria infections here are attributed to *Plasmodium falciparum*, 2.02% to *Plasmodium malariae* and 0.68% to *Plasmodium ovale* [41]. Three study sites were selected along an altitudinal transect, ranging from 750 to 1,965 m above sea level (Figure 1).

The village of Santchou (5°15′N; 9°50′E) with 37,479 inhabitants [42], is located at an altitude of 750 m within the wide Mbô plain, whose landscape is a shrubby savannah with some isolated woodland along the streams, limited towards north-east by a slopy forest cliff at about 14 km from Santchou. The river Nkam and its tributaries provide a dense hydrography and the swampy plain floods frequently during the rainy seasons. The annual average temperature is 28 ± 3°C with daily thermal amplitude of less than 10°C. Rains are abundant with annual average of 2,200 mm.

On top of this sheer cliff, at an altitude of 1,400 m on the Bamiléké plateau, lies the city of Dschang (5°27′N; 10°04′E) with 120,207 inhabitants [42], located at 22 km from Santchou. The topography is characterized by the juxtaposition of small hills furrowed by little streams flowing down towards swampy lakes. The average annual temperature is 20.5 ± 6°C, with February being the hottest month. The daily thermal amplitude can exceed 13°C during the dry season, and constitutes the peculiarity of this locality. The mean annual rainfall is 2,000 mm.

Finally, Djuttitsa (5°36′N; 10°05′E) a village of Nkongni District with 53,367 inhabitants [42], is located at approximately 20 km away from Dschang towards the mountains at an altitude of 1,965 m. The village is surrounded by: an industrial tea plantation, the experimental land of IRAD and a wide pasture for cattle. The average annual temperature is 16.5 ± 7°C and the mean annual rainfall is 1,600 mm.

### Field study design and blood sampling

Longitudinal parasitological surveys were conducted once a week and during 12 months, in three sentinel health centres namely: Santchou District Hospital, Hôpital Saint Vincent de Paul of the catholic mission Secret Heart of Dschang and the Djuttitsa District Hospital, located in the above described localities. Participants were selected among outpatients living in these localities, based on the malaria-like complaints, which have been recorded. The following parameters were also determined: age and axillary temperature (assuming a case definition of fever to be any temperature ≥38°C). Blood samples were collected by finger pricking, then thin and thick blood films were made on the same slide for microscopic examinations.

### Laboratory examination of slides

The dried slides were brought back to the laboratory where they were fixed by dipping the thin film into the May-Grünwald solution for five seconds and washed with physiological water (0.9% of NaCl). Then both the thick and thin films were stained with 10% Giemsa for 15 minutes [43]. Examination of thick blood films enabled the determination of the presence and density of malaria parasites, while thin blood films were used to identify the *Plasmodium* species [44].

A thick blood film was declared negative if no parasite (regardless the stage) was found after examining at least 200 microscope fields [43]. For the positive slides, two consecutive methods were used to count malaria parasites in thick blood films: the semi-quantitative system (or “+” system) for quick forward of the result to the physician in charge and a rapid treatment with artemether-lumefantrine (Coartem®), followed by a quantitative evaluation, where the parasitaemia was calculated for research purpose, by counting parasites against 1,000 WBC [44].

### Statistical analyses

Data were entered into Microsoft office excel 2007 and transferred into SPSS.18 for windows. The Chi-square test was used to compare: the rate of occurrence of each symptom between *Plasmodium*-positive and *Plasmodium*-negative patient groups, as well as the rate of *Plasmodium* infections, and gametocyte prevalence among population ages and study sites. When in a given locality the difference in the rate of occurrence of a symptom between *Plasmodium*-positive and *Plasmodium*-negative patients group was significant, it was assumed that there is a link between the carriage of *Plasmodium* parasite and the fact of having that symptom at 0.05 level of significance (Table 1). The syndrome was then shifted from “malaria-like” to “malaria-related”, and taken into consideration in the definition of febrile episode (temperature ≥ 38°C or reported fever during the preceding days, plus some associated malaria-related symptoms or signs mentioned in Table 1), and hence selected for the calculation of MAFE.

**Table 1:** **Comparison of the occurrence rate of syndromes in** ***Plasmodium*** **positive and** ***Plasmodium*** **negative patient groups**

**Prevalence of signs and symptoms at the different study sites of the altitudinal transect**									
**Syndromes**	**Santchou (750 m)**			**Dschang (1400 m)**			**Djuttitsa (1965 m)**		
	**PPP (No P)**	**PNP (No P)**	**Comparison**	**PPP (No P)**	**PNP (No P)**	**Comparison**	**PPP (No P)**	**PNP (No P)**	**Comparison**
Fever*	71.18	49.63	*χ* ^2^ = 25.23	74.94	40.21	*χ* ^2^ = 15.33	74.43	30.22	*χ* ^2^ = 11 .23
			p ≤ 10^−5^			p ≤ 10^−5^			p ≤ 10^−5^
Average temperature	38.6 (341)	36.7 (336)	t = 9.76	38.7	37,3	t = 11.76	39.2 (169)	37,1 (567)	t = 13.02
			p = 0.04			p = 0.02			p = 0.01
			(SG)	(966)	(1462)	(SG)			(SG)
Headache*	36.7 (245)	12.5 (432)	*χ* ^2^ = 54.83	19.90 (608)	1.70 (1820)	*χ* ^2^ = 257.19	31.91 (141)	3.53 (595)	*χ* ^2^ = 112.51
			p ≤ 10^−7^			p ≤ 10^−7^			p ≤ 10^−7^
			(SG)			(SG)			(SG)
Joint pains*	27.35 (245)	13.89 (432)	*χ* ^2^ = 18.58	26.32 (608)	1.81 (1820)	*χ* ^2^ = 373.95	31.21 (141)	4.20 (595)	*χ* ^2^ = 97.84
			p ≤ 10^−5^			p ≤ 10^−7^			p ≤ 10^−7^
			(SG)			(SG)			(SG)
Abdominal pains*	19.18 (245)	49.07 (432)	*χ* ^2^ = 11.07	24.34 (608)	3.79 (1820)	*χ* ^2^ = 236.50	17.73 (141)	6.39 (595)	*χ* ^2^ = 18.74
			p ≤ 10^−4^			p ≤ 10^−7^			p ≤ 10^−5^
			(SG)			(SG)			(SG)
Convulsion*	4.08 (245)	0.93 (432)	*χ* ^2^ = 7.69	0.66 (608)	0.05 (1820)	*χ* ^2^ = 8.06	1.42 (141)	0.34 (595)	*χ* ^2^ = 7.69
			p ≤ 10^−3^			p ≤ 0.02			p ≤ 10^−3^
			(SG)			(SG)			(SG)
Nausea*	6.53 (245)	2.78 (432)	*χ* ^2^ = 4.66	0.99 (608)	0.22 (1820)	*χ* ^2^ = 6.54	0.71 (141)	0.34 (595)	*χ* ^2^ = 4.66
			p ≤ 0.02			p ≤ 0.02			p ≤ 0.02
			(SG)			(SG)			(SG)
Asthenia	23.27 (245)	20.37 (432)	*χ* ^2^ = 0.78	8.22 (608)	1.65 (1820)	*χ* ^2^ = 61.84	13.48 (141)	2.02 (595)	*χ* ^2^ = 37.09
			p ≤ 0.38			p ≤ 10^−7^			p ≤ 10^−7^
			(NS)			(SG)			(SG)
Anorexia	20.00 (245)	18.52 (432)	*χ* ^2^ = 0.22	18.09 (608)	1.92 5 (1820)	*χ* ^2^ = 212.19	11.35 (141)	2.02 (595)	*χ* ^2^ = 27.12
			p ≤ 0.64			p ≤ 10^−7^			p ≤ 10^−7^
			(NS)			(SG)			(SG)
Constipation	1.22 (245)	7.17 (432)	*χ* ^2^ = 11.61	1.64 (608)	0.82 (1820)	*χ* ^2^ = 3.01	2.13 (141)	0.34 (595)	*χ* ^2^ = 5.42
			p ≤ 10^−4^			p ≤ 0.08			p ≤ 10^−4^
			(SG)			(NS)			(SG)
Diarrhoea	2.45 (245)	16.90 (432)	*χ* ^2^ = 31.67	1.15 (608)	1.00 (1820)	*χ* ^2^ = 0.12	2.13 (141)	2.52 (595)	*χ* ^2^ = 31.67
			p ≤ 10^−7^			p ≤ 0.73			p ≤ 10^−7^
			(SG)			(NS)			(SG)
Vomiting	4.90 (245)	7.41 (432)	*χ* ^2^ = 1.62	3.29 (608)	1.00 (1820)	*χ* ^2^ = 15.66	1.42 (141)	2.69 (595)	*χ* ^2^ = 1.62
			p ≤ 0.20			p ≤ 10^−5^			p ≤ 0.20
			(NS)			(SG)			(NS)
Itches	0.41 (245)	2.31 (432)	*χ* ^2^ = 3.56	0.80 (608)	0.38 (18200	*χ* ^2^ = 0.04	0.71 (141)	0.50 (595)	*χ* ^2^ = 3.56
			p ≤ 0.06			p ≤ 0.85			p ≤ 0.06
			(NS)			(NS)			(NS)
Dizziness	1.63 (245)	3.70 (4320)	*χ* ^2^ = 2.34	2.80 (608)	0.77 (1820)	*χ* ^2^ = 14.85	2.13 (141)	0.50 (595)	*χ* ^2^ = 2.34
			p ≤ 0.13			p ≤ 10^−4^			p ≤ 0.13
			(NS)			(SG)			(NS)

PPP = % of *Plasmodium* positive patients, PNP = % of *Plasmodium* negative patients, No P = Number of patients examined, SG = significant, NS = not significant, * = malaria related manifestation in all the 3 sites.

The parametric Student t–test was used to compare the mean temperature among *Plasmodium* infected and non-infected patient groups in the three different localities, and 95% confidence intervals were constructed. Differences in the mean parasitaemia (asexual and sexual), among seasons and between population ages in each locality were tested using the non-parametric Kruskal Wallis H-test. The difference between the prevalence and means were declared significant when the probability p was < 0.05.

### Ethical issues

An institutional ethical approval (N°: 0287-05/SG/CAB, dated 17/03/2005) was obtained from OCEAC, followed by a national ethical clearance (N°: FWA IRB00001954, dated 11/05/2005) delivered by the National Ethics Committee (Yaoundé, Cameroon). The various aspects of the work were conducted in collaboration with the local Health District Authorities. The free and informed consent of patients was requested through individual discussions and group meetings, between the sentinel team and the patients. For those willing to cooperate, a written informed consent was obtained prior to their enrolment in the study. Free malaria treatment with artemether-lumefantrine (Coartem®) was given to all patients referred to the team in case of *Plasmodium* infection as recommended by the National Malaria Control Programme.

## Results

### *Plasmodium* infection rate and malaria infectious reservoir along the transect

A total of 48 surveys were carried out on the outpatients attending the health centres of the three above described sites, and a total of 677, 2428, and 736 patients were examined in the lowland plain, the highland plateau and uphill respectively. Table 2 shows the rate of *Plasmodium* infected subjects as well as the densities of asexual and sexual parasites at each altitudinal level. The prevalence of overall *Plasmodium* infections showed a significant reduction along the altitudinal transect (*χ*^2^ = 59.52, p =10^−8^). Also, comparison of *Plasmodium* densities in the three different localities with Kruskal Wallis test showed a decrease (H =2.0, df =2, p =0.007).

**Table 2:** ***Plasmodium falciparum*** **parasite rate (PfPR) and density among the outpatients attending health centres in the three altitudinal study sites**

		***Plasmodium falciparum*** infections*			
**Localities (Altitude)**	**Number of outpatients examined**	**Asexual parasite prevalence**	**Sexual parasite prevalence**	**Mixed infections**	**Overall parasite prevalence**
		**(MD ± SD)**	**(MD ± SD)**	**(No of patients)**	**(MD ± SD)**
Santchou (750 m)	677	36.19%^a^	2.07%^a^	1.03%	38.26%^a^
		(14569 ± 1786)	(25.4 ± 5.5)	(n =7)	(14597 ± 2407)
Dschang (1400 m)	2428	25.04%^b^	0.45% ^a^	0.33%	25.49%^b^
		(11553 ± 1548)	(44.0 ± 3.2)	(n =8)	(11599 ± 1868)
Djuttitsa (1965 m)	736	19.16%^c^	1.08% ^a^	0.00%	20.24%^c^
		(6234 ± 1425)	(26.0 ± 2.9)	(n =0)	(6262 ± 1125)
Total	3841	25.88%	0.86%	0.39%	26.74%
		(14954 ± 3835)	(29.4 ± 3.7)	(n =15)	(14983 ± 3845)

MD: Mean density, SD: Standard deviation.

*: Numbers in the same column sharing different letter superscripts (a, b or c) differ significantly (p < 0.05).

In distinguishing the parasite stages, it was observed that both the prevalence and the density of asexual parasites decreased with increasing altitude from; 36.19% (14,569/μl of blood), to 25.04% (11,553/μl) and 19.16% (6,234/μl), in Santchou, Dschang and Djuttitsa, respectively. Statistical analysis of the asexual parasite prevalence by Chi-square test showed a highly significant reduction of the prevalence with increasing altitude (*χ*^2^ = 55.74, df =2, p =10^−8^). Likewise comparing the mean density of trophozoites by H test of Kruskal Wallis showed significant reduction with increasing altitude (H =86.16, df =2, p =10^−3^). Concerning the sexual stages, the human malaria infectious reservoir (HMIR) was 2.07% (25.4 gametocytes/μl) in lowland, 0.45% (44.0 gametocytes/μl) in the highland plateau and 1.08% (26.0 gametocytes/μl) at the hill site, showing no significant difference of the HMIR between the three sites (*χ*^2^ = 59.55, p =0.91 for the prevalence; and H =2.00, df =2, p =0.37 for the density).

### Malaria syndrome

Table 1 compares the occurrence rate of syndromes in *Plasmodium*-positive and *Plasmodium-*negative patient groups. Fever was found to be a characteristic symptom of malaria along the altitudinal transect, since both the rate of febrile patients and the mean temperature were significantly higher in the group of *Plasmodium* positive patients compared to the *Plasmodium* negative group. Five additional manifestations were simultaneously related to malaria in the three sites: headache, joint pains, abdominal pains, convulsion and nausea. However, there was a heterogeneity in the other malaria syndromes along the transect.

### Distribution of *Plasmodium* infections among population ages at each altitudinal level

**Figure 2:**
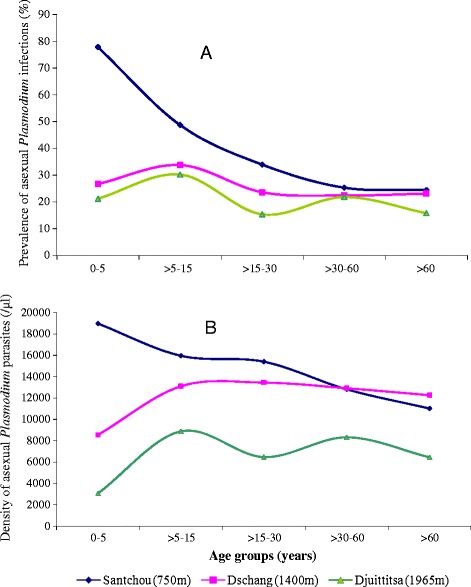
**Distribution of asexual** ***Plasmodium*** **infections among population ages at each altitudinal level (A: Prevalence, B: Mean density).**

Figure 2A shows the distribution of prevalence of asexual malaria parasites among population ages at each study site. The rate of *Plasmodium* infection peaked in children less than five years of age in Santchou, where transmission is higher [40], then there was a significant reduction in the prevalence of infections from the children to the adult age groups (*χ*^2^ = 44.25, p =10^−8^). In Dschang, the prevalence showed no variation in age groups (*χ*^2^ = 13.67 df =4, p =0.084). Likewise in Djuttitsa, this prevalence was almost similar for all the age groups (*χ*^2^ = 7.87, df =4, p =0.097).

The mean of asexual parasite densities also peaked in children less than five years of age in Santchou (Figure 2B) and decline from the younger age group to the older ones, leading to a significant reduction in the trophozoite density (Kruskal Wallis test, H =33.71, df =4, p =10^−3^). However, in Dschang as well as in Djuttitsa, the mean parasite densities were fairly similar for all the age groups (Kruskal Wallis test, H =11.84, p =0.09 for Dschang; H =8.62, p =0.07 for Djuttitsa).

**Figure 3:**
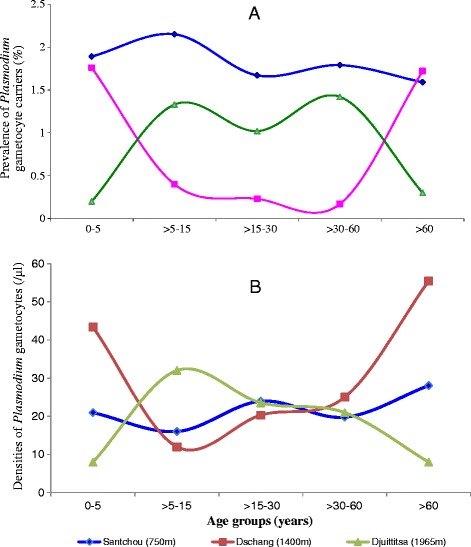
**Distribution of sexual** ***Plasmodium*** **infections among population ages at each altitudinal level (A: Prevalence, B: Mean density)** **.**

The proportion of visits with patent gametocytes in the Mbô plain of Santchou did not show any significant difference with age (Figure 3A), despite the fact that malaria incidence here was higher in the childhood age group, and all the age groups displayed gametocyte carriers (*χ*^2^ = 6.08, p =0.20). However, in Dschang and Djuttitsa, the prevalence of gametocyte carriers was constant only within the age range of 5 to 60 years. On the plateau site, gametocyte index was higher in the extreme age groups (0 – 5 and >60) as compared to 5 – 60 age group (*χ*^2^ = 6.04, df =1, p =0.008). Conversely uphill the gametocyte index was lower in these extreme age groups (*χ*^2^ = 4.19, df =1, p =0.008).

Figure 3B shows that the mean densities of *Plasmodium* gametocytes among population ages in Santchou did not differ significantly. On the plateau and uphill, the pattern of sexual parasite density among age groups was similar to the prevalence. In fact, there was no difference in the gametocyte densities among the age groups of 5 to 60 years. However, on the plateau site, gametocyte density was higher in the extreme age groups (0 – 5 and >60) as compared to 5 – 60 age groups (H =6.06, df =1, p =0.019); conversely uphill the gametocyte density was lower in these extreme age groups (H =3.12, df =1, p =0.005).

### Seasonal variations of *Plasmodium* infection and human malaria infectious reservoir

**Figure 4:**
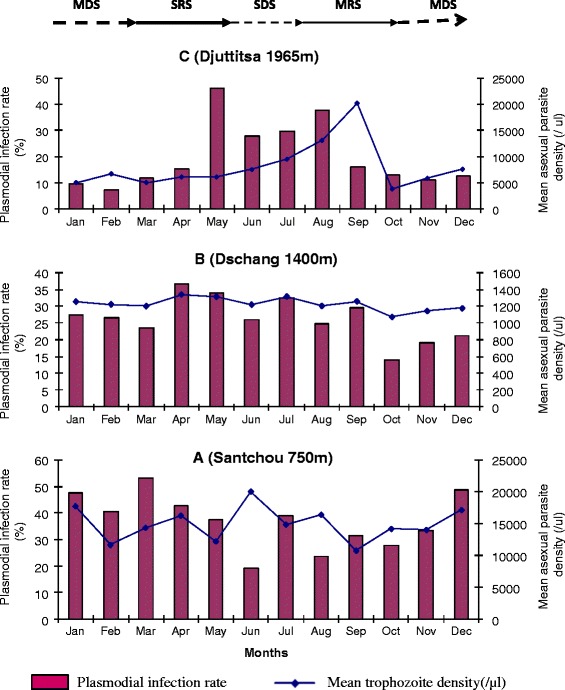
**Seasonal variations of asexual** ***Plasmodium*** **infection rate and mean density in Santchou (A), Dschang (B) and Djuttitsa (C).** MDS: main dry season, SRS: short rainy season, SDS: short dry season and MRS: main rainy season.

Figure 4 shows the seasonal variations of the infection rate and parasitaemia along the transect. In Santchou and Dschang, the prevalence of infections and the malaria parasite densities were perennial throughout the year (Figure 4A and B) and both plasmodial rate and parasitaemia did not show any real peak. Statistical analysis of the prevalence showed that there was no difference between the months (*χ*^2^ = 25.22, p =0.51), as well as between seasons (*χ*^2^ = 25.56, p =0.11). Comparison of parasitaemia with Kruskal Wallis test came to the same conclusion: H =29.40, df =11, p =0.12 between months, and H =16.13, df = 3, p =0.09 between seasons. On the contrary, the prevalence of malaria infections in Djuttitsa was seasonal and occurred from May to August (mainly during the SDS); while the parasite density peaked in September (Figure 4C). Statistical test showed that there was a significant difference in the prevalence of infections and in the parasite densities of the different seasons; *χ*^2^ = 30.01, df =3, p =10^−6^ for prevalence, and H =54.56, p =10^−3^ for parasitaemia.

**Figure 5:**
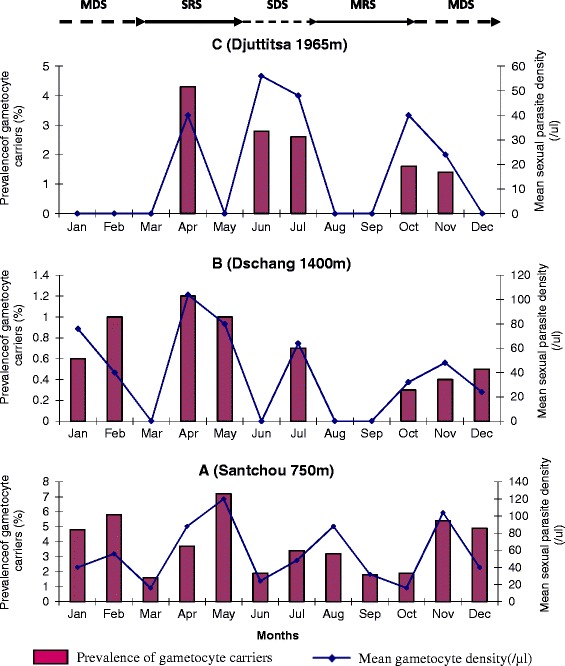
**Seasonal variations of** ***Plasmodium*** **gametocyte carriers prevalence and mean gametocytaemia in Santchou (A), Dschang (B) and Djuttitsa (C).** MDS: main dry season, SRS: short rainy season, SDS: short dry season and MRS: main rainy season.

Figure 5 describes the seasonal variations of sexual *Plasmodium* across the transect and it did not display any typical patterns. Comparison with Fisher exact test and Kruskal Wallis test showed no seasonal variation in the gametocyte carriers prevalence and in gametocyte densities respectively, at each altitudinal site along the transect. The striking observation here was the absence of gametocyte carriers in the highland area during the peak period of the trophozoite density (i.e. August to September).

### The fraction of *Plasmodium*-infected patients among the symptomatic subjects

When considering only the symptomatic patients attending the health centres (i.e. febrile subjects with temperature ≥38°C or reported fever during the preceding days, eventually plus the other associated malaria symptoms or signs mentioned in Table 1), a total of 341 (50.37%), 966 (39.78%) and 169 (22.96%) patients were enrolled in the lowland plain, the highland plateau and at the hill site respectively. Table 3 gives the rates of *Plasmodium-*infected subjects (PfPR) among the symptomatic populations along the altitudinal transect. This malaria-attributable fraction of fever episodes (MAFE) represented 77.07% in the lowland at Santchou, 75.81% in Dschang and 78.13% in Djuttitsa. There was a significant reduction in the percentage of symptomatic cases among outpatients consulting in health centres with the altitudinal climate variation (*χ*^2^ = 117.10, p =10^−8^). However comparing the rate of *Plasmodium*-infected patients among the febrile individuals (MAFE) showed no significant difference along the altitudinal transect (*χ*^2^ = 59.52, p =0.18).

**Table 3:** **The fraction of** ***Plasmodium***-**infected patients among the symptomatic populations (MAFE) attending the health centres along the altitudinal transect**

**Localities (Altitude)**	**Number of symptomatic patients (%)**	***Plasmodium*** **infections among the febrile outpatients examined**			
		**Asexual parasite prevalence**	**Sexual parasite prevalence**	**Mixed infections**	Overall parasite prevalence (MAFE)*
		**(MD ± SD)**	**(MD ± SD)**	**(No of patients)**	**(MD ± SD)**
Santchou (750 m)	341^a^	71.18%	8.21%	2.32%	77.07%^a^
	(50.37%)^a^	(14569 ± 11786)	(25.43 ± 5.49)	(n =8)	(14597 ± 8407)
Dschang (1400 m)	966^b^	74.94%	1.14%	0.21%	75.81%^a^
	(39.78%)^b^	(11553 ± 1548)	(44.00 ± 3.23)	(n =2)	(11599 ± 10868)
Djuttitsa (1965 m)	169^c^	74.43%	4.7%	0.00%	78.13%^a^
	(22.96%)^c^	(6234 ± 11425)	(26.00 ± 2.91)	(n =0)	(6262 ± 8125)
Total	1476	73.89%	3.18%	0.68%	77.07%
	(38.43%)	(14954 ± 13835)	(29.36 ± 3.72)	(n =10)	(14983 ± 13845)

MD: Mean density, SD: Standard deviation.

*The numbers in the same column sharing different letter superscripts (a, b or c) differ significantly (p < 0.05).

## Discussion

### *Plasmodium* infections among the overall outpatients attending the health centres

The *P. falciparum* parasite rate (PfPR) used in this study to compare malaria incidence along the transect is a commonly reported index in malaria epidemiology. In fact, it is historically consistent with the classical categories of malaria endemicity (hypo-, meso- and hyper-endemic) and, therefore, useful for standardization; moreover, it has good biological, epidemiological and statistical properties [45]. In this study, the authors went further and discriminated between the asexual and sexual parasites. There was a drop in the asexual *Plasmodium* prevalence and density among the presumptive malaria cases recorded, with increasing altitude. Considering the classification of malaria zones into endemic categories according to Metsslaar and Van Thiel, Santchou presenting a prevalence of 36.19% is in a mesoendemic malaria zone, Dschang with 25,04% is an intermediate situation, while Djuttitsa with a prevalence of 19.16% is hypoendemic. This PfPR continuous decrement with altitude was already observed in Tanzania [46-48]. It may be indirectly due to the relief, the climate and the agricultural landscape as mentioned in Kenya by [49]. In fact, the higher prevalence of *Plasmodium* infection in Santchou was also a consequence of the high number of anopheline’s breeding sites. This number was higher in lowland because the hillside gradients provide efficient drainage, resulting in numerous and variable potential breeding sites rather in lowland areas [50]. Moreover, the flatness of the Mbô plain is a factor which favours mosquito breeding and thereby transmission.

Surprisingly, malaria prevalence at Djuttitsa was higher than expected from the entomological inoculation rate previously determined [40]. This discrepancy may be due to human migration. In fact, people of Dschang do go for farming at Djuttitsa where the soil is fertile, while those of Djuttitsa move down to Dschang and even Santchou frequently for trading. Migration has already been shown to influence the epidemiology of highland malaria in Burundi, where parasite prevalence appears to be high with a very low transmission [51]. The observation is also supported in Western Kenya by [52], who stipulated that travelling from highland to lowland is a significant risk factor for malaria attack.

Since the prevalence and density of malaria infection decrease with increasing altitude while the gametocyte rate and density do not vary, the scarcity of malaria disease in highlands is likely due to the absence of vectors and not the parasites. In fact, the highland climate is known to reduce the survivorship and reproduction fitness of anopheline mosquitoes [1,40]. In case of climate change towards global warming, there might be a sudden increase in anopheline population size; since the infectious reservoir is furnished and the human population of highlands lacks immunity [2], the village will then be prone to a malaria outbreak. Findings that climate variability plays an important role in initiating malaria epidemic were already observed in Kenya [53] and in North-Eastern Tanzania [38,54]. Fortunately, no outbreak was observed during the study period. If that has been the case, it is conceivable that the incidence of febrile malaria episodes at these highland sites, would have suddenly exceeded the one found even in normal holoendemic villages, and the physicians with the sentinel workers of this project could not have coped with the high number of patients. Such an epidemic situation was recently experienced by the population of the Far North region of Cameroon (Maroua and several other villages of the Mandara mountain); during this malaria outbreak from September to November 2013 (with a peak in October) about 187,000 malaria cases with some 979 deaths were recorded [55].

### Malaria-related manifestations

As in most endemic localities of sub-Saharan Africa, several other manifestations were related to malaria, in addition to fever, as the most prominent sign of malaria. However, these signs and symptoms are not specific to malaria as mentioned elsewhere [28,38], because malaria shares with many other infectious diseases syndromes that are clinically similar, as a consequence of having the same pathogenic pathways in common [27]. Moreover, these manifestations vary from one locality to another [56,57]. Altitudinal climate variations in the outskirts and fringes of the Bamiléké plateau in Western-Cameroon provided an opportunity to verify and confirm this hypothesis. Fever, headache, joint pains, abdominal pains, convulsion and nausea were malaria-related manifestations regardless the altitude; while the link of other symptoms with malaria depended upon the site. The conclusion drawn here is that, to minimize the risk of wrong clinical diagnosis, the signs and symptoms should be related to altitude in the algorithm of malaria treatment.

### Distribution of *Plasmodium* infections among population ages

Asexual parasite prevalence and density decrease with age after an initial peak in the 0–5 years age group in the lowland plain of Santchou; meanwhile in Dschang and Djuttitsa where transmission is low, prevalence and density were similar in all age groups. Similar high prevalence in younger age groups have been reported in studies elsewhere in Africa, as in Kenya [58], in Tanzania [59,60] and in several other localities [61]. The high burden of malaria borne by the children of Santchou may be due to the fact that they were not yet immune at a younger age. The decline in the burden with age is due to progressive acquired immunity, while the similar parasite prevalence and density in all the age groups in Dschang and Djuttitsa is attributable to the fact that no immunity is acquired as the entomological inoculation rate (EIR) is low, causing the whole age groups of the population to be equally susceptible to infections. In fact, an entomological survey in these sites had indicated that the EIR decreases from the plain to the highland [40].

The gametocyte prevalence and density did not show any variation with age in the Mbô plain of Santchou; however some discrepancy was noted on the plateau and uphill, with extreme age groups (<5 and > 60 years), which represent the non-active age range of the population. It was observed that this age range in Dschang has the tendency of a higher gametocyte index. Conversely, non-active inhabitants of the highland have the trend to lack gametocytes. The conclusion drawn here is that inhabitants of the Bamileké Plateau who are sedentary are exposed to a higher risk of malaria infection, whereas people staying uphill must migrate down to be in contact with malaria parasite. This is in conformity with previous findings indicating that the EIR at Djuttitsa is null [40]. In fact, active individuals of Dschang do go for farming at Djuttitsa where the volcanic soil is fertile and therefore stay away from mosquitoes for some time, while those of Djuttitsa come down to Dschang and even Santchou for trading and come therefore in contact with mosquito bites. Migration has already been shown to influence the epidemiology of highland malaria in Burundi where parasite prevalence also appears to be high with a very low transmission [51]. This result was also observed in Western Kenya [52], where travelling from highland to lowland was a significant risk factor for malaria attack.

### Seasonal variations of *Plasmodium* infection rates

In the lowland plain of Santchou and on the Bamiléké plateau, *Plasmodium* infection was perennial throughout the year, but seasonal uphill, with malaria episodes occurring from May to August (i.e. mostly during the SDS), probably due to the abundance of puddles and the alleviation of climate constraints (temperature and relative humidity) on mosquito breeding and survival during the preceding SRS. During the malaria period, which coincides with the peak biting rate of anopheline mosquitoes [40], there was a continuous increase of the mean parasite density in the human population of highlands, which reached the peak in September. The striking observation here is the absence of gametocyte carriers in the highland area during the peak period of trophozoite density (August to September); a phenomenon which was also observed on the Plateau in Dschang, suggesting absence of malaria parasite circulation during the preceding season. In fact, migration of populations up- and down-wards is seasonal. This seasonal pattern increases the risk of malaria epidemic in case of climate change towards global warming, which is likely to favour a sudden mass production of mosquitoes. Since the infectious reservoir is furnished, there might be a quick spreading of malaria into highland fringes from the lowland as already suggested [62]. This is the reason why it was advocated that local malaria control strategies in low transmission settings should also include interventions that aim to reduce the gametocyte carriage in the population [63]. Strategies such as intermittent preventive treatment during the period from May to August could be of interest in this area.

### Malaria-attributable fraction estimate among febrile subjects attending the health centres

Malaria-attributable fraction (which is based on the case definition of malaria) is very complex to determine in endemic areas, due to: asymptomatic carriage of malaria parasites and non-malarial fevers, which introduce some bias. In fact, estimates of MAFE should define parasite densities threshold to classify a fever episode as related or not to malaria. In this respect, logistic regression method with model fever risk as continuous function of parasite density was explored, and gave more precise estimates than simple analyses of parasite prevalence [64]. In addition to the better precision, it also defines the relation between parasite density and fever, and how this varies with age and region [65].

Herein, a unique and simple method was tested along an altitudinal transect. The malaria-attributable fraction was considered to be the PfPR among the fever episodes, where fever episodes refer to subjects with temperature ≥ 38°C (or reported fever during the preceding days), plus some associated malaria-related symptoms or signs determined in Table 1. The threshold density was defined as being the patent one detectable using the microscopic method described. It was so adopted (regardless the altitude and the age of patients) to take into account de fact that in highlands, there is no protective immunity, and very low *Plasmodium* density can cause severe disease at all ages [4]. Furthermore, a parameter was needed whose sudden change in the highland value would create a discrepancy on the altitudinal transect, and constitute an alert for the MEWS. To bridge the gap of this low density threshold, some malaria-related symptoms were associate to the definition of “fever episode” (Table 1).

After putting the same sampling effort in all of the three sites, a high number of fever episodes were detected in the lowland plain as compared to the highland, but with the same *Plasmodium* infection rates (malaria fractions). The comment raise is that the higher number of infective bites found in lowland would result in only low infection in human populations due to natural immunity, while in highlands, though there is no immunity, there is also low infective bites due to the climate which is unfavourable to the *Plasmodium* extrinsic cycle, the anopheline development and their reproductive fitness. It is likely therefore that in the lowland, there are much more pathogens that can cause fever apart from *Plasmodium,* which is not the case in highland areas. These other pathogens were responsible for 12.11% (50.37 – 38.26) of fever episodes, 14.29% (39.78 – 25.49) and 2.72% (22.96 – 20.24), in Santchou, Dschang and Djuttitsa respectively (Tables 2 and 3). Consequently, in this latter zone of low endemicity, the malaria parasite’s presence in fever cases can be considered to be malaria disease incidence. Nevertheless, significant progress in the control of malaria has been made and led to a call for laboratory diagnosis-based treatment strategies of fever episodes where it is possible [35,36,66], in replacement of the WHO report [35], stating that for all febrile episodes, presumptive treatment with artemisinin-based combination therapy should be administered. From the above mentioned results, it is obvious that this change in the treatment policy is not necessary in highlands.

Since the MAFE as well as the HMIR do not vary with altitude, they may be reliable parameters to detect incipient malaria outbreaks in highlands. In fact, it is obvious that an incipient malaria outbreak in the highland would raise their levels in highlands as compared to lowlands. Therefore, in addition to guiding the diagnostic strategies, they can also be used to predict and forecast malaria epidemics.

## Conclusion

Evidence for the operational feasibility of using the HMIR and the MAFE as indicators in a MEWS was shown. It is recommended that in designing the MEWS in Cameroon, the National Malaria Control Programme should include the HMIR and the MAFE as indicators to be taken into account by a network of health workers in sentinel stations. They should put special emphasis on: altitude of the localities and age groups, and intensify control before and during the SDS, in order to better prevent and forecast an eventual malaria epidemic. Implementation, monitoring and evaluation of this strategy in the sentinel health centres will help confirming the usefulness and cost-effectiveness of these indicators in case a malaria outbreak occur.
